# The Association between *icaA* and *icaB* Genes, Antibiotic Resistance and Biofilm Formation in Clinical Isolates of Staphylococci spp.

**DOI:** 10.3390/antibiotics11030389

**Published:** 2022-03-15

**Authors:** Seham Abdel-Shafi, Heba El-Serwy, Yehia El-Zawahry, Maysaa Zaki, Basel Sitohy, Mahmoud Sitohy

**Affiliations:** 1 Botany and Microbiology Department, Faculty of Science, Zagazig University, Zagazig 44519, Egypt; hegazyseham@yahoo.com (S.A.-S.); hebaelserwy@yahoo.com (H.E.-S.); yehiaelzawahry@gmail.com (Y.E.-Z.); 2 Clinical Pathology Department, Faculty of Medicine, Mansoura University, Mansoura 35516, Egypt; maysaazaki5@hotmail.com; 3 Department of Clinical Microbiology, Infection and Immunology, Umeå University, SE-90185 Umeå, Sweden; 4 Department of Radiation Sciences, Oncology, Umeå University, SE-90185 Umeå, Sweden; 5 Biochemistry Department, Faculty of Agriculture, Zagazig University, Zagazig 44511, Egypt

**Keywords:** *Staphylococcus aureus*, biofilm, *icaA*, *icaB*, microtiter plate assay, multidrug resistance

## Abstract

Sixty-six (66) *Staphylococcus* bacterial isolates were withdrawn from separate clinical samples of hospitalized patients with various clinical infections. Conventional bacteriological tests identified the species of all isolates, and standard microbiological techniques differentiated them into CoPS or CoNS. Their biofilm development was followed by an analysis via the MTP (microtiter tissue culture plates) technique, and we then investigated the presence/absence of *icaA* and *icaB*, which were qualified in the top-30 potent biofilm-forming isolates. Thirteen isolates (46.7%) showed the presence of one gene, six (20%) isolates exhibited the two genes, while ten (33.3%) had neither of them. The formation of staphylococci biofilms in the absence of *ica* genes may be related to the presence of other biofilm formation *ica*-independent mechanisms. CoPS was the most abundant species among the total population. *S. aureus* was the sole representative of CoPS, while *S. epidermidis* was the most abundant form of CoNS. Antibiotic resistance was developing against the most frequently used antimicrobial drugs, while vancomycin was the least-resisted drug. The totality of the strong and medium-strength film-forming isolates represented the majority proportion (80%) of the total investigated clinical samples. The biochemical pattern CoPS is associated with antibiotic resistance and biofilm formation and can be an alarming indicator of potential antibiotic resistance.

## 1. Introduction

Generally, the Gram-positive staphylococci are the leading cause of device-related infections (DRIs). Among the staphylococci, *Staphylococcus aureus* is the focus of most clinical concerns since its infections are commonly more severe and aggressive than those caused by other Staphylococci spp. due to its vast and diverse arsenal of aggressive toxins and virulence factors. Next to *S. aureus*, *Staphylococcus epidermidis*, a less aggressive skin commensal, has drawn attention as a frequent cause of biofilm-associated infection from medical devices and associated complications, including bloodstream infections [1]. The species of *Staphylococci* were distinguished into two groups based on their ability to coagulate blood: coagulase-positive *Staphylococci* (CoPS), where *Staphylococcus aureus* is the most important, and coagulase-negative staphylococci (CoNS), which comprises the majority of other species, including *S. epidermidis*. The species *S. aureus* is a dangerous human pathogen, causing opportunistic infections and severe life-threatening diseases, such as severe sepsis, pneumonia, toxic shock syndrome and endocarditis. Additionally, some pathogenic strains of *S. aureus* are resistant to the antibiotic methicillin (methicillin-resistant *S. aureus* (MRSA)), engendering a critical issue in hospitals [2].

The production of biofilms is a conservative process, enabling bacterial survival under challenging environmental conditions. However, unwanted biofilm accumulation may lead to many undesirable impacts, such as the biofouling of heat alteration systems and pelagic structures, microbial deterioration of metal sheeting, dental decay and home contamination of food and medications, as well as short- and long-term biomedical implants and devices [3]. Staphylococci are the most frequent source of biofilm-related infections, being a common pathogen of human skin and mucosal surfaces and a variety of other animals, more evidently and distinctly than other biofilm-associated pathogens. In addition, when implanted during an operation, staphylococci may represent a potentially infectious agent contaminating the surgical equipment that access these regions [4]. The importance of biofilm production in the pathogenesis of *S. aureus* and the development of multidrug-resistant bacterial strains (MDR strains) has been documented [5].

A polysaccharide intercellular adhesin (PIA) encoded within *ica* operon was concluded to be an essential factor for staphylococcal biofilm formation, among other adhesive factors [6]. The locus of intercellular adhesion of *ica* consists of a four-gene operon (*ica ABCD*), *encoding* the essential proteins necessary for PIA production. The first two genes, *icaA* and *icaB*, play primary roles in exopolysaccharide synthesis [7]. The *icaA* gene encodes N-acetylglucosaminyl-transferase, while *icaB* encodes PIA de-acetylase [7,8].

The purpose of this study was to delineate the biofilm-forming ability of some pathogenic isolates of staphylococci within a random population of medical samples, using a quantitative microtiter plate assay. Simultaneously, the presence or absence of the *icaA* and *icaB* genes was assessed via PCR in strong biofilm-forming staphylococci, confirmed using the MTP method, to establish the potential association between the two. Establishing this association may suggest that these genes can be used as biomarkers in screening Staphylococci for their potential biofilm formation and potential antibiotic resistance.

## 2. Materials and Methods

### 2.1. Bacterial Isolates

This study was conducted within the Mansoura Faculty of Medicine Ethical Committee (ethical approval code: R.20. 10.15). A prospective cross-sectional study was performed in Mansoura University Hospital, Egypt, from February 2019 to February 2020. A total of sixty-six (66) bacterial isolates of staphylococci were withdrawn from separate clinical samples of hospitalized patients with various clinical infections, collected from different medical departments, i.e., Allergy and Immunity, Blood Diseases, Cardiology, Contagious Diseases and Malnutrition, Endocrine Glands and Sugar, External Section, Heart disease, ICU, Nephrology, Neurology, Surgery Care and The Care of Novices.

Conventional bacteriological tests identified all isolates. All the samples were inoculated into blood agar (BA) and MacConkey agar (MA), and the isolates were identified as CoPS or CoNS using standard microbiological techniques, including biochemical, slide and tube coagulase tests [9]. The bacterial isolates were maintained at −70 °C until being investigated. 

### 2.2. Species Identification of Clinical Isolates

Staphylococci isolates were identified utilizing Siemens Healthcare Diagnostics’ MicroScan WalkAway-96 Systeme International d’Unites (Pos ID Type 2) panels. This system was developed for in vitro diagnostic analysis to identify the species level of aerobic and facultative Gram-positive *Staphylococci*.

### 2.3. Antibiotic Susceptibility Assessment

Antibiotics susceptibility was conducted using a disc diffusion method complying with the Clinical and Laboratory Standards Institute guidelines (CLSI) [10]. The discs used for antibiotic susceptibility were vancomycin (VA) 30 μg, amoxicillin–clavulanate (Aug) 25 μg, erythromycin 20 μg, trimethoprim–sulfamethoxazole (SXT) 25 μg, imipenem (IPM) 10 μg, oxacillin (OX) 1 μg, ceftriaxone (CAX) 30 μg and clindamycin (CLI).

### 2.4. Studying Biofilm Formation via Microtiter Plate (MTP)

A crystal violet staining assay determined all *Staphylococcus* isolates’ capacity to form biofilms [11]. An uninoculated medium was used as a negative control to determine the background OD. The typical OD values were calculated for all tested strains and negative controls, and therefore the cut-off OD value (ODc) was established. For interpreting the results, strains were divided into the following groups: 

(I) OD ≤ ODc = no biofilm producer, (II), ODc < OD ≤ (2 × ODc) = weak biofilm producer, (III) (2 × ODc) < OD ≤ (4 × ODc) = moderate biofilm producer, (IV) (4 × ODc) < OD = strong biofilm producer [12].

### 2.5. Detection of icaA and icaB Genes

#### 2.5.1. Genomic DNA Extraction

For extracting the genomic DNA of an isolate, several colonies were mixed and suspended in an aliquot of lysis buffer (20 μL) containing 0.25% SDS, 0.05 N NaOH. The suspension was heated at 95 °C for 7 min, cooled on an ice bath and centrifuged briefly at 16,000× *g* for 2 min. The supernatant was diluted by adding 180 μL of distilled water and centrifuging for 5 min at 16,000× *g* to remove cell debris. The final supernatant was used as the source template for DNA amplification.

#### 2.5.2. PCR

Strong biofilm-forming *Staphylococcus* species were selected for molecular screening for *icaA* and *icaB* using PCR. The genes *icaA* and *icaB* were amplified using PCR in staphylococci strains to identify amplicons of 188 bp and 190 bp utilizing reference primers [13,14]. An Eppendorf master cycler^®^, MA thermocycler (Hamburg, Germany) was used for DNA amplification. The running program consisted of 30 denatured cycles at 94 °C for 60 s, 55 °C for 60 s of annealing time and 72 °C for 60 s of expansion, with the final 10 min at 72 °C. The *icaB* amplification included five minutes of initial denaturation at 94 °C, 30 cycles of denaturation at 94 °C over 60 s, 30 s of annealing at 52 °C and 10 min of final extension at 72 °C over 90 sec. [13]. After electrophoresis on a 1% agarose gel, a UV transilluminator (Bio-Rad, Watford, United Kingdom, WD17) visualized the PCR products.

### 2.6. Statistical Analysis

All statistical studies utilized SPSS statistical analysis software (SPSS, Inc., Chicago, IL, USA) version 22. The significance of the obtained results was judged at the 0.05 level. The Chi-square test was used for comparing two or more groups.

## 3. Results

### 3.1. Bacterial Isolates and Relative Distribution

This study included 66 *Staphylococcus* isolates extracted from various biological materials, such as blood (72.2%, 48/66), urine (12.1%, 8/12.1), pus (9.1%, 6/66), tube (3%, 2/66), drain (1.5%, 1/66) and abscess (1.5%, 1/66). Table 1 represents the distribution of *Staphylococcus* spp. isolated from patients. The information indicates that CoPS, represented by *Staphylococcus aureus*, was isolated from 46 patients accounting for about 70% of the total, i.e., the majority. On the other hand, CoNS isolates were collected from 20 patients, representing about 30% of the total. The species *S. epidermidis* was the dominant species in the isolates of CoNS, representing about 9% of the total (Table 1). Finally, *S. aureus* was the most dominant of all detected *Staphylococcus* spp. among the studied isolates (70%) and the sole CoPS that was represented, while *S. epidermidis* was the second most dominant one (9%), followed by *S. hyicus* (6%), within the CoNS group.

### 3.2. Antibiotic Resistance Distribution

The studied isolates (66) were divided into two groups based on their sensitivity to different antibiotics and classified into CoPS or CoNS isolate biochemical patterns on the basis of standard microbiological techniques, including biochemical, slide and tube coagulase tests [9].

The data in Table 2 revealed that the antibiotic resistance developed more pronouncedly among the CoNS isolates, exhibiting more than 90% resistance against four antibiotics, i.e., amoxicillin–clavulanate, imipenem, oxacillin and ceftriaxone. A similar magnitude of resistance (more than 90%) was noticed within CoPS against only one antibiotic (oxacillin). Therefore, the resistance developed against oxacillin is highly spread among all the studied isolates, whether CoPS or CoNS. Within the CoPS group, the highest resistance was developed against oxacillin (93.5%), followed by clindamycin (76.1%), then ceftriaxone and erythromycin (60.9%). On the other hand, the least resistance to antibiotics is noticed against vancomycin, recording only 24% and 15% resistance in isolates among CoPS and CoNS, respectively. Generally, antibiotic resistance developed against most of the frequently used antimicrobial drugs, while vancomycin was the least-resisted antibiotic, where the recorded resistance was about 24 and 15% within the CoPS and CoNS isolates, respectively.

### 3.3. Detection of the Biofilm Formation via Microtiter Tissue Culture Plates

The microtiter plates assay (MTP) outcomes (Table 3) indicate that each staphylococci isolate of the tested samples exhibited attachment at different extents. Thirty isolates out of the total tested isolates (66 isolates), representing 45.5% of the total, strongly produced biofilm on MTP. These strong biofilm-forming isolates comprised 19 CoPS isolates and 11 CoNS isolates.

Strong and moderate film formation was more evident in CoNS than CoPS, occurring in about 55 and 40% (totaling 95%) of the investigated CoNS isolates against 41.3 and 32.6% (totaling 74%) of the CoPS isolates. There was only one weak biofilm-forming isolate within the CoNS group against twelve isolates in the CoPS group, representing 5 and 26% of the respective total isolates in each group.

Generally, the total number of strong and medium biofilm-forming isolates represented the majority, amounting to about 80% of the total investigated clinical samples with relatively more frequency in the CoNS than the CoPS type.

### 3.4. Detection of icaA and icaB Genes

**Figure 1:**
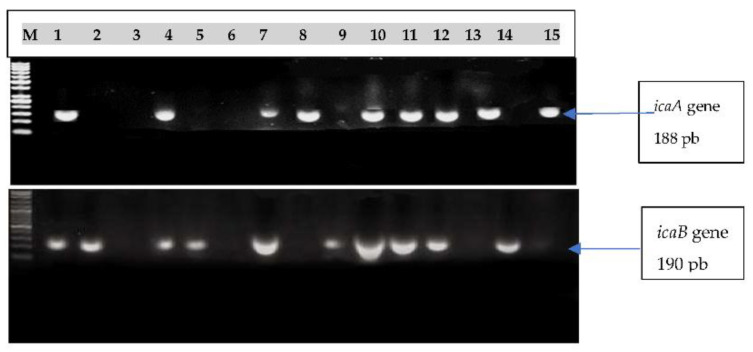
Electrophoretic pattern of Multiplex PCR amplicons of *icaA* gene and *icaB* gene on agarose gel in 15 samples of the 30 investigated staphylococci isolates (Table 4). Lane M was a 50 bp DNA ladder.

The 30 strong biofilm-forming isolates were tested for the presence of *icaA* or *icaB* genes via molecular screening using PCR amplification, and the visualized amplicons are shown in Figure 1. The *icaA* gene appeared in 9 isolates, while *icaB* appeared in 10 out of 15 investigated isolates.

The results in Table 4 and Table 5 indicate that, among the selected thirty strong-film forming isolates, six isolates, representing 20% of the total, contained both of the two *ica* genes (*icaA* and *icaB*) and 14 isolates had one of the *ica* genes (*icaA* or *icaB*). Conclusively, 20 out of 30 isolates, i.e., 66.6% of the total, were positive for either genes or both. On the other hand, 10 out of 30 isolates (33.3% of the total), were negative for both genes (*icaA* or *icaB*). Alternatively, the *icaA* gene was positive in nine (47%) CoPS isolates while only in one CoNS isolate (9%). In total, this gene was present in 10 isolates, representing about 33% of selected staphylococci species. Consequently, *icaA* was absent in most of the tested isolates, i.e., 10 CoPS and 10 CoNS, representing together about 52.6 and 90.9% of the total, respectively.

The second gene (*icaB*) was relatively more detected in both CoPS and CoNS isolates, i.e., 10 and 6 isolates, respectively, representing 53.3% of the total. As a result, the absence of this gene was noticed in nine and five isolates of CoPS and CoNS, respectively. These represented about 47.4 and 45.5% of the total, respectively. Furthermore, the amplified *icaA* and *icaB* regions were detected on agarose gel electrophoresis at 188 and 190 bp, respectively (Figure 1). Finally, the majority (66.7%) of the studied strong biofilm-forming isolates indicated the presence of both *icaA* or *icaB* genes or one of them, while a still considerable portion (33.3%) of isolates did not contain any of these genes. Additionally, *icaB* gene could be noticed as more associated with strong biofilm formation in the two groups of staphylococci species (CoPS and CoNS).

### 3.5. The Association between Antibiotic Resistance, Biofilm Formation and the Biochemical Pattern

The biochemical pattern (CoPS or CoNS) was analyzed in the investigated antibiotic-resistant isolates and the biofilm-forming ones (Table 6). It can be observed that a CoPS pattern was prevalent in most antibiotic-resistant isolates. It reached more than 65% of the isolates resistant to most studied antibiotics (six out of eight studied antibiotics). The maximum prevalence (79%) of CoPS pattern was witnessed in the vancomycin-resistant isolates. The isolates resistant to amoxicillin–clavulanate and imipenem exhibited less presence of CoPS pattern (51 and 45%, respectively). The relatively higher prevalence of CoPS in the antibiotic-resistant isolates was also observed in all biofilm-forming isolates, with either strong, medium or weak biofilm forming, indicating a high association between the two phenomena, irrespective of the degree of biofilm formation.

## 4. Discussion

*S. aureus* was generally qualified as the most efficient bacterium in shielding itself against host antagonistic reactions through the production of biofilms. Biofilms can limit the bacterial clearance by antimicrobial agents and host immunological responses [15], and thus enables bacteria to invade host tissue, forming metastatic abscess growth leading to morbidity and mortality [16]. Sepsis caused by *Staphylococcus aureus* in cancer patients represents a substantial cause of morbidity and mortality in these patients [17].

The current analysis of 66 medical isolates revealed the presence of 46 isolates (69.7%) as CoPS, represented uniquely by *S. aureus*, and the presence of 20 medical isolates (30.3%) as CoNS, where *S. epidermidis* was the prevalent species. A similar outcome was noted by [18,19]. Hence, the spread of this type is complying with the global spreading pattern of this type (CoPS) and this species (*S. aureus*) without apparent impact from the geographical site.

A significant relationship between the biofilm type and the development of biofilms was reported in prior studies [20]. In the United States, a somewhat more significant proportion of biofilm-positive bacteria isolated from non-fluid locations, such as superficial/deep skin, bone, and the respiratory tract, than from host fluids, such as blood or urine, was seen in clinical trials [20]. However, in the current study, most of the investigated samples were fluids, i.e., 48 blood, 8 urine, 6 puss, 2 tubes and 1 drain. The biofilm formation phenomenon was prevalent and variably distributed among strong, intermediate and weak biofilm producers (data not shown). Most isolates belonged to the strong and intermediate biofilm producers; their sum reached 62 and 75% of blood and urine samples, respectively. The relatively higher proportion of strong and intermediate bio-film producers may agree with [21], who reported a higher incidence of urinary isolates in biofilms than in other clinical sample isolates.

A high degree of resistance against the frequently used antibiotics, e.g., oxacillin and ceftriaxone, clindamycin and erythromycin, has been noticed in the current study, indicating limited antimicrobial choices against staphylococci isolates. A lower degree of resistance was noticed against vancomycin. Comparable effects were also demonstrated by [22]. Although vancomycin was the least susceptible to microbial resistance, considerable proportions of developed resistance among CoPS and CoNS isolates amounting to about 24 and 15%, respectively, were recorded. Similar intermediate resistance of staphylococci isolates against vancomycin was previously reported [23]. This alarming report and the current findings should urgently motivate more research before we lose the race.

New proteins and peptides have been found to be effective antimicrobial agents [23,24,25] and can substitute the currently resisted antibiotics. Some new peptides and proteins were comparable or more effective than the current antibiotics in in vitro studies [26,27] and needed to be in vivo tested and validated. The relatively higher effectiveness of vancomycin against different pathogens may be partially due to its chemical nature as a glycopeptide, containing a carbohydrate moiety in its structure. Recently, a glycoprotein from catfish (CFG) exhibited considerable antibacterial activity. Combining CFG with specific antibiotics at equal ratios induced synergistic antibacterial actions [28]. Hence, these glycoproteins may be the target for generating new antibacterial agents capable of counteracting the developed antibiotic resistance.

The MTP assay method is the most reliable, precise and reproducible approach for determining biofilm development by the clinical isolates from staphylococci. It has the benefit of being a quantitative approach to compare compliance with various strains [29]. The current study results indicated that strong and medium biofilm-forming isolates represented the majority and amounted to about 80% of the total investigated clinical samples with a relatively greater frequency of having a CoPS biochemical pattern [30], with a relatively lower frequency of biofilm formation in staphylococcal clinical isolates, amounting to 57.8%. There is a strong association between biofilm formation and the resistance developed against the frequently used antibiotics [31,32]. Hence, this high proportion of biofilm formation may explain considerable resistance against most frequently used antibiotics.

In our study, *icaA* and *icaB* were found in 33% and 53% of selected isolates, respectively, while Al-Mtory et al. (2016) demonstrated that the prevalence of *icaA* and icaB was 95.8% and 91.6%, respectively [33]. The study of Diemond-Hernández et al. (2010) detected *icaA* in 10.3% but did not detect the *icaB* gene [34]. Our study did not identify all tested *ica* operon genes in the tested strains, in agreement with previous reports [34,35].

The statistical test results did not indicate a link between *icaA* and *icaB* genes with the formation of biofilms in selected Staphylococci spp. These results may mean that in staphylococci, biofilm formation is determined not only by the expression of biofilm genes, i.e., the genes *icaA* and *icaB*, but also by other factors inherent in the medium composition, e.g., salt content, iron and anaerobic atmosphere may be influential [36]. Although most of the studied strong biofilm-forming isolates indicated the presence of both *icaA* and *icaB* genes or one of them, a still considerable portion (33.3%) of isolates did not contain any of these genes. This result may refer to the presence of additional biofilm formation mechanisms that are not necessarily *ica*-dependent. Therefore, the research for these potentially unknown mechanisms may open the way for discovering new appropriate drugs against these pathogens. However, further studies using different clinical samples and techniques may be needed to confirm the results that have so far been obtained.

The prevalence of the biochemical pattern CoPS in the investigated antibiotic-resistant isolates in most antibiotic-resistance strains may indicate that this pattern helps in establishing antibiotic resistance. The high prevalence (65%) of the isolates resistant to most studied antibiotics may indicate the importance of this biochemical activity in establishing antibiotic resistance. Furthermore, since the biochemical type CoPS was highly prevalent (79%) in biofilm-forming vancomycin-resistant isolates, it may also be involved in developing antibiotic resistance and can be employed as an alarming indicator of potential antibiotic resistance. Additionally, the prevalence of CoPS in all biofilm-forming isolates irrespective of their degree of film formation may refer to a high association between this biochemical pattern and biofilm formation.

## 5. Conclusions

CoPS are the most abundant species among the total population of the medical samples, where S. aureus was the sole representative of this group. In the second order comes *S. epidermidis*, which is the most abundant species of CoNS. Antibiotic resistance is developing against most frequently used antimicrobial drugs, while vancomycin was the least-resisted antibiotic. However, the relatively small resistance (15–24%) against vancomycin necessitates an urgent search for other effective antibiotics or antimicrobial agents. The totality of the strong and medium biofilm-forming isolates represented a majority proportion, amounting to about 80% of the total investigated clinical samples with relatively more frequency in the CoNS than the CoPS type. The majority (66.7%) of the studied strong biofilm-forming isolates indicated the presence of both *icaA* and *icaB* genes or one of them, while a still considerable portion (33.3%) of isolates did not contain any of them. Additionally, the *icaB* gene could be noticed as more associated with strong biofilm formation in the two groups of staphylococci species (CoPS and CoNS). This result may refer to the presence of additional biofilm formation mechanisms that are not necessarily ica-dependent. Therefore, research into these potentially unknown mechanisms may help us find the appropriate drugs for combatting these pathogens. The prevalence of biochemical pattern CoPS in antibiotic-resistant isolates may indicate some association between antibiotic resistance and biofilm formation. CoPS can serve as an alarming indicator of potential antibiotic resistance. Further studies using different clinical samples and different techniques may be needed to confirm the thus-far obtained results and conclusions.

## Figures and Tables

**Table 1:** Relative distribution of *Staphylococcus* spp. isolates collected from patients.

Bacterial Type	*Staphylococcus* spp.	No.	%
**CoPS**		46	69.7
	*S. aureus*	46	69.7
**CoNS**		20	30.3
	*S. epidermidis*	6	9.1
	*S. hyicus*	4	6.1
	*S. hominis*	3	4.5
	*S. xylosus*	3	4.5
	*S. haemolyticus*	2	3.0
	*S. auricularies*	1	1.5
	*S. sciuri*	1	1.5
**Total**		**66**	**100**

**CoPS:** Coagulase-positive staphylococci, represented only by *S. aureus.* **CoNS:** Coagulase-negative staphylococci, represented by 7 species of staphylococci.

**Table 2:** Mapping resistance extent (%) within the *Staphylococcus* isolates collected from patients.

Type of Antibiotics		CoPS		CoNS		*p* Value
	Code	No	%	No	%	%
Vancomycin	1	11	23.9	3	15.0	χ^2^ = 0.663 *p* = 0.416
Amoxicillin–clavulanate	2	20	43.5	19	95.0	χ^2^ = 15.31 *p* < 0.001 *
Erythromycin	3	28	60.9	15	75.0	χ^2^ = 1.23 *p* = 0.268
Trimethoprim–sulfamethoxazole	4	21	45.7	8	40.0	χ^2^ = 0.181 *p* = 0.671
Imipenem	5	15	32.6	18	90.0	χ^2^ = 0.181 *p* < 0.001 *
Oxacillin	6	43	93.5	18	90.0	χ^2^ = 0.241 *p* = 0.624
Ceftriaxone	7	28	60.9	19	95.0	χ^2^ = 7.92 *p* = 0.005 *
Clindamycin	8	35	76.1	10	50.0	χ^2^ = 4.37 *p* = 0.037 *

* Statistically significant (at *p* < 0.05) using Chi-square test.

**Table 3:** Biofilm formation, estimated via microtiter tissue culture plates, amongst 66 bacterial isolates collected from patients and classified into 46 CoPS and 20 CoNS isolates.

Biofilm Formation	CoPS		CoNS		*p*-Value
	No.	%	No.	%	
Strong	19	41.3	11	55	0.304
Moderate	15	32.6	8	40	0.562
Weak	12	26.1	1	5	0.047 *
Total	46	100	20	100	

Chi-square test; * statistically significant (at *p* < 0.05). CoPS is represented only by S. aureus, while CoNS is represented by 7 species of staphylococci.

**Table 4:** Distribution of (*icaA*) and (*icaB*) genes in the strong biofilm-forming isolates within the tested *Staphylococcus* species isolated from patients, classified as strong biofilm-forming isolates.

No. of Isolates	Tested Isolates	*icaA* Gene	*icaB* Gene
1	*S. aureus*	+	+
2	*S. aureus*	−	+
4	*S. aureus*	−	−
6	*S. aureus*	+	+
7	*S. aureus*	−	+
8	*S. aureus*	−	−
9	*S. aureus*	+	+
11	*S. aureus*	+	−
12	*S. aureus*	−	+
16	*S. aureus*	+	+
18	*S. aureus*	+	+
21	*S. aureus*	+	+
25	*S. aureus*	+	−
41	*S. aureus*	−	+
42	*S. aureus*	+	−
43	*S. aureus*	−	−
44	*S. aureus*	−	−
45	*S. aureus*	−	−
46	*S. aureus*	−	−
47	*S. sciuri*	−	+
48	*S. hominis*	−	+
52	*S. haemolyticus*	+	−
54	*S. hominis*	−	−
55	*S. xylosus*	−	+
56	*S. hyicus*	−	+
57	*S. hyicus*	−	+
58	*S. hyicus*	−	−
59	*S. xylosus*	−	+
60	*S. epidermidis*	−	−
63	*S. auricularies*	−	−

**Table 5:** Relative detectability of *icaA* and *icaB* genes in PCR results within the tested staphylococci species isolated from patients.

Category	Isolate Code				Isolate Quantity		
					No.		%
**Two *ica* gene**	1, 6, 9, 16, 18, 21.				6		20
**One *ica* gene**	2, 7, 11, 12, 25, 41, 42, 47, 48,52, 55, 56, 57, 59.				14		46.7
**No *ica* gene**	4, 8, 43, 44, 45, 46, 54, 58, 60, 63.				10		33.3
	**CoPS (*n* = 19)**		**CoNS (*n* = 11)**		**Total (*n* = 30)**		***p* Value**
***icaA ***	**No.**	**%**	**No.**	**%**	**No.**	**%**	
Positive	9	47.4	1	9.1	10	33.3	**χ^2^ = 4.59** *p* = 0.032 *
Negative	10	52.6	10	90.9	20	66.7	
*icaB*							**χ^2^ = 0.01** ***p* = 0.919**
Positive	10	52.60%	6	54.50%	16	53.30%	
Negative	9	47.40%	5	45.50%	14	46.70%	

Used test: Chi-square test; * statistically significant (at *p* < 0.05). CoPS is represented only by *S. aureus*, while CoNS is represented by 7 species of staphylococci.

**Table 6:** The association between antibiotic resistance, biofilm formation and biochemical pattern (CoPS or CoNS) in Staphylococci spp. isolates collected from medical samples.

Antibiotic	Antibiotic Resistance		Biofilm Formation									% CoPS *
			Strong			Medium			Weak			
	Quantity of Isolates					No. of Isolates						
	Total No	%	Total No	CoPS	CoNS	Total No	CoPS	CoNS	Total No	CoPS	CoNS	
Vancomycin	14	21	8	**6**	2	5	**4**	1	1	**1**	0	**79**
Amox/clav	39	59	19	8	11	14	7	7	6	5	1	51
Erythpomycin	43	65	17	**11**	6	17	**9**	8	9	**8**	1	**65**
Trimeth/sulf	29	44	10	**6**	4	12	**8**	4	7	**7**	0	**72**
Imipenem	33	50	17	7	10	14	7	7	2	1	1	45
Oxacillin	61	92	28	**18**	10	22	**15**	7	11	**10**	1	**70**
Ceftriaxone	47	71	20	**13**	5	18	**15**	4	9	**8**	0	**77**
Clindamycin	45	68	19	**10**	9	18	**13**	6	8	**8**	1	**69**

* Total no. of CoPS isolates in biofilm-forming isolates/total no. of antibiotic resistant isolates. The bold numbers indicate the prevalence of CoPS in comparison with CoNS.

## Data Availability

Not applicable.
